# Factors associated with the length of stay in total knee arthroplasty patients with the enhanced recovery after surgery model

**DOI:** 10.1186/s13018-019-1389-1

**Published:** 2019-11-06

**Authors:** Guoqing Li, Jian Weng, Chang Xu, Deli Wang, Ao Xiong, Hui Zeng

**Affiliations:** 1 National & Local Joint Engineering Research Center of Orthopaedic Biomaterials, Peking University Shenzhen Hospital, Shenzhen, People’s Republic of China; 2 Department of Bone & Joint Surgery, Peking University Shenzhen Hospital, Shenzhen, China

**Keywords:** Enhanced recovery after surgery, Total knee arthroplasty, Length of stay, Factors

## Abstract

**Objectives:**

The purpose of this study is to identify the factors that influence the length of stay (LOS) in total knee arthroplasty (TKA) patients with an enhanced recovery after surgery (ERAS) program.

**Methods:**

Information from 167 patients (31 males and 136 females, range from 43 years to 88 years old) who underwent the unilateral elective primary TKA from January 2017 to January 2019 were reviewed retrospectively. Factors were analyzed by single-factor variance and multi-factor linear regression.

**Results:**

By single-factor variance analysis, American Society of Anesthesiologists (ASA) physical status classification system, pre-operation albumin, pre-operation erythrocyte sedimentation rate (ESR), primary and merge diseases, hidden blood loss, and length of operation were correlated with LOS (*P* < 0.05). Multi-factor linear regression results suggested that gender, ASA class, pre-operation Alb, and pre-operation ESR were associated with LOS (*P* < 0.05). Moreover, ASA class 3 (*B* value 4.84), pre-operation Alb < 30 g/L (*B* value 18.33), and pre-operation ESR > 15 mmol/h (*B* value 2.21) could increase the LOS, while males (*B* value − 3.56) had a shortened LOS.

**Conclusions:**

Overall, our research found that female, ASA class 3, pre-operation Alb < 30 g/L, and pre-operation ESR > 15 mmol/h could extend LOS in TKA patients with ERAS.

## Introduction

End-stage knee diseases could cause patients great inconvenience, and the population of these patients increases continuously [1]. Total knee arthroplasty (TKA) is an effective treatment for end-stage knee diseases, and it had been matured with decades of development and continuous research. Enhanced recovery after surgery (ERAS) has made great success for patients after TKA, which shortens the recovery time and improves patients’ satisfaction. However, factors that might extend the length of stay (LOS) of patients remained to be identified [2–4]. In our study, we aim to investigate factors affecting the LOS of patients with ERAS after TKA treatment using a retrospective analysis.

## Materials and methods

### Applications of ERAS model

**Fig. 1:**
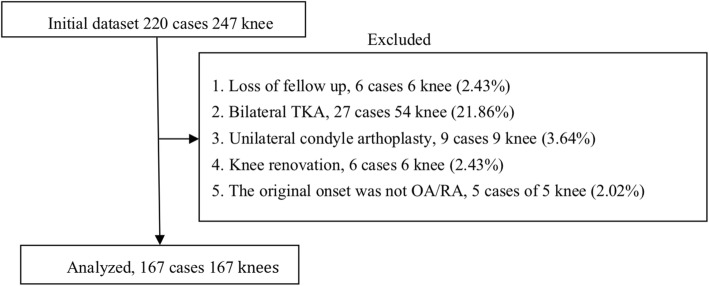
Flowchart that illustrates patients excluded from the study

ERAS was proposed by Kehlet et al. [5] firstly. It can decrease the surgical stress and complications, shorten the LOS, and improve patient satisfaction. Edwards et al. reported that the ERAS model was safe and effective for elderly patients older than 80 years [6]. Previously, our group developed the relevant diagnosis and treatment plan for patients with end-stage knee diseases, according to the ERAS consensus guide for joint arthroplasty in China [7]. A retrospective analysis of the Institutional Joint Registry was performed to identify factors affecting the LOS of patients. The study protocol was approved by the Human Subject Committee at Ethics Committee of Peking University Shenzhen Hospital (Ethics Committee of Peking University Shenzhen Hospital (research) [2019] 38th). The investigation of the LOS of TKA was performed carefully to ensure the validity. Patients who were lost follow-up, who underwent bilateral TKA in the same period, unilateral condyle arthroplasty, or renovation, and had primary diseases without osteoarthritis/rheumatoid arthritis (OA/RA) were excluded. These data were collected retrospectively from the medical records. In total, 167 patients who underwent surgery in the Department of Bone and Joint in the Peking University Shenzhen Hospital were enrolled in this study (Fig. 1).

### Study variables

Variables analyzed in the study included the following: (1) demographic characteristics: age, gender, mass, height, and body mass index (BMI); (2) preoperative blood test: hemoglobin (Hb), albumin, C-reactive protein (CRP), and ESR; (3) postoperative blood test: Hb, Alb, CRP, ESR, ASA class, and operative time; (4) transfusion and change of hemoglobin (△Hb); (5) the use of urinary duct, tourniquet, and incision drainage, primary diseases, and merge diseases; and (6) date of admission and discharge.

### Discharge criteria

The discharge criteria vary worldwide [8]. The discharge criteria for patients after TKA in our hospitals are as follows: (1) patients are willing to be discharged; (2) patients’ vital signs are normal and stable, while the performance of appetite and sleep has returned to normal; (3) the incision has healed with no signs of infection, and hematological results are in the normal range; (4) patients have passed the functional exercise test, and they can walk alone with the help of mobility aids; (5) visual pain score ≤ 3; and (6) active knee joint flexor ≥ 90°, passive flexural ≥ 110°, and muscle strength grade ≥ 4.

### Statistical analysis

All statistical analyses were carried out by SPSS software (version 23.0). The data is represented by mean ± standard deviation. The single factor adopted the chi-square test, and the multi-factor adopted the linear regression analysis. *P* < 0.05 is considered statistically significant.

## Results

### Single-factor chi-square analysis

The single-factor chi-square analysis of demographic characteristics and LOS showed that demographic characteristics, including sex, age, and BMI, had no significant correlation with LOS (*P* > 0.05) (Table 1).

**Table 1:** Single-factor chi-square analysis of the demographic characteristics (*N* = 167)

Factors	Classification	Number	Percent	Mean ± SD	*F* value	*P* value
Gender	Female	136	81.4	19.24 ± 6.72	0.275	0.601
	Male	31	18.60	18.55 ± 6.37		
Age (year)	Y < 50	6	3.60	19.83 ± 3.60	0.540	0.706
	50 ≤ Y < 60	22	13.20	19.09 ± 4.51		
	60 ≤ Y < 70	74	44.30	18.32 ± 7.25		
	70 ≤ Y < 80	53	31.70	20.02 ± 6.45		
	80 ≤ Y	12	7.20	19.67 ± 8.15		
BMI (kg/m^2^)	BMI < 18.5	3	1.80	19.67 ± 5.51	0.620	0.685
	18.5 ≤ BMI < 24	59	35.30	19.05 ± 7.08		
	24 ≤ BMI < 28	63	37.70	19.49 ± 7.17		
	28 ≤ BMI < 30	20	12.00	17.65 ± 5.32		
	30 ≤ BMI < 40	19	11.40	18.68 ± 2.89		
	40 ≤ BMI	3	1.80	24.33 ± 13.01		

*BMI* body mass index

Some items in the preoperative blood tests were related with the LOS. Preoperative Alb, preoperative ESR, primary disease, merge disease, and ASA class were associated with LOS. The variation was statistically significant (*P* < 0.05). At the same time, preoperative Hb and preoperative CRP had no significant correlation with LOS (*P* > 0.05) (Table 2).

**Table 2:** Single-factor chi-square analysis of preoperative variables (*N* = 167)

Factors	Classification	Number	Percent	Mean ± SD	*F* value	*P* value
Pre-operation Hb (g/L)	Hb < 100	5	3.00	20.00 ± 5.79	5.457	0.104
	100 ≤ Hb < 110	13	7.80	22.08 ± 10.17		
	110 ≤ Hb < 120	23	13.80	21.74 ± 7.09		
	120 ≤ Hb < 130	52	31.10	17.29 ± 4.68		
	130 ≤ Hb < 140	46	27.50	18.87 ± 7.60		
	140 ≤ Hb	28	16.80	19.21 ± 4.98		
Pre-operation albumin (g/L)	< 30	2	1.20	33.50 ± 4.95	4.675	0.002
	≥ 30	165	98.80	18.94 ± 6.48		
Pre-operation ESR (mm/h)	< 15	77	46.10	17.71 ± 5.22	6.560	0.011
	≥ 15	90	53.90	20.31 ± 7.47		
Pre-operation CRP (mg/L)	< 5	44	26.30	18.55 ± 6.48	0.371	0.691
	≥ 5	122	73.70	19.29 ± 6.73		
Primary disease	RA	16	9.60	23.50 ± 7.80	8.050	0.005
	OA	151	90.40	18.65 ± 6.36		
Merge disease	No	112	67.10	18.36 ± 5.96	4.509	0.035
	Yes	55	32.90	20.65 ± 7.68		
ASA class	1	38	22.80	16.61 ± 5.17	3.844	0.023
	2	120	71.90	19.75 ± 6.53		
	3	9	5.40	21.22 ± 10.76		

*Hb* hemoglobin, *ESR* erythrocyte sedimentation, *CRP* C-reactive protein, *ASA* American Society of Anesthesiologists

The hidden blood loss (pre-operation Hb) and the operation duration in surgical factors were statistically correlated with LOS for TKA patients, and there was a significant difference (*P* < 0.05). However, the start time of operation, intraoperative blood transfusion, the use of urinary duct, tourniquet, and incision drainage tube had no significant correlation with LOS (*P* > 0.05) (Table 3).

**Table 3:** Single-factor chi-square analysis of operational variables (*N* = 167)

Factors	Classification	Number	Percent	Mean ± SD	*F* value	*P* value
Hidden blood loss (△Hb) (g/L)	△Hb < 10	30	18.00	19.30 ± 6.56	2.729	0.031
	10 ≤ △Hb < 20	56	33.50	20.77 ± 8.23		
	20 ≤ △Hb < 30	43	25.70	17.84 ± 4.54		
	30 ≤ △Hb < 40	32	19.20	18.94 ± 5.55		
	40 ≤ △Hb	6	3.60	12.83 ± 3.66		
Surgery begins	< 14:00 P.M.	53	31.70	18.86 ± 6.60	0.525	0.470
	≥ 14:00 P.M.	114	68.30	19.66 ± 6.75		
Op-duration T (h)	T < 2	24	13.50	17.92 ± 3.96	4.524	0.002
	2 ≤ T < 3	97	55.90	18.38 ± 5.49		
	3 ≤ T < 4	35	25.20	23.00 ± 9.78		
	4 ≤ T < 5	3	1.40	15.33 ± 2.08		
	5 ≤ T	8	4.00	16.00 ± 3.21		
Transfusion	No	152	91.00	18.82 ± 6.15	2.682	0.071
	Yes	15	9.00	22.07 ± 10.25		
Urine tube	No	33	19.80	17.70 ± 6.46	1.882	0.172
	Yes	134	80.20	19.46 ± 6.67		
Drainage	No	147	88.00	19.09 ± 6.56	8.345	0.894
	Yes	20	12.00	19.30 ± 7.36		
Tourniquet	No	2	1.20	12.50 ± 3.54	2.021	0.157
	Yes	165	98.80	19.19 ± 6.63		

**Fig. 2:**
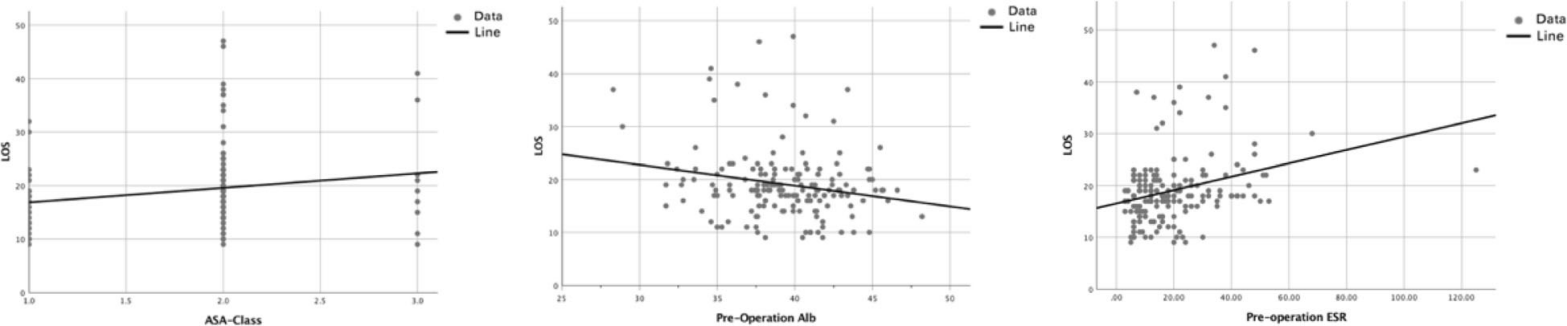
Regression curves

The influence of pre-operation ESR, ASA class, and pre-operation Alb on the LOS in TKA patients was reflected in regression curves (Fig. 2). Among these variables, ASA class and preoperative ESR had a positive correlation with LOS of patients, while preoperative Alb had a negative correlation with LOS of patients.

Moreover, the results of single-factor variance analysis in this study suggested that some factors were significantly associated with LOS (*P* < 0.05), such as preoperative Alb, preoperative ESR, primary disease, merge disease, and ASA class. Combining the reported literatures and the clinical practice, factors that showed no statistical significance in the single-factor variance analysis (*P* > 0.05) are included in the multi-factor linear regression analysis.

### Multi-factor linear regression analysis

The results suggested that gender, ASA class, preoperative Alb, and preoperative ESR were significantly correlated with LOS in TKA patients (*P* < 0.05). Moreover, female patients, ASA class 3, pre-operation albumin < 30 g/L, and pre-operation ESR > 15 mmol/h were the risk factors for the prolongation of LOS in TKA patients, while the other factors were not significantly correlated with LOS (*P* > 0.05) (Table 4).

**Table 4:** Multi-factor linear regression analysis (*N* = 167)

Factors	*β*	*S.E*	*T* value	*P* value	95%CI
Gender (*X*_1_)					
Female	(Control group)				
Male	− 3.56	1.58	− 2.25	0.03	− 6.69~− 0.42
Age (*X*_2_)					
Y < 50	(Control group)				
50 ≤ Y < 60	2.80	3.30	0.85	0.40	− 3.72~9.34
60 ≤ Y < 70	0.26	3.33	0.08	0.94	− 6.32~6.86
70 ≤ Y < 80	− 0.82	3.36	− 0.24	0.81	− 7.46~5.83
80 ≤ Y	− 0.67	3.88	− 0.17	0.86	− 8.35~7.01
ASA class (*X*_3_)					
1	(Control group)				
2	2.49	1.36	1.83	0.07	− 0.21~5.18
3	4.84	2.51	1.92	0.049	− 0.14~9.81
Duration T (*X*_4_)					
T < 2H	(Control group)				
2H ≤ T < 3H	1.07	2.13	0.50	0.62	− 3.16~5.29
2H ≤ T < 3H	8.09	2.86	2.84	0.005	2.44~13.74
4H ≤ T < 5H	5.26	6.61	0.80	0.43	− 7.83~18.35
T ≥ 5H	4.71	5.74	0.82	0.41	− 6.67~16.09
Pre-operation albumin (*X*_5_)					
≥ 30 g/L	(Control group)				
< 30 g/L	18.33	5.41	3.39	0.001	7.61~29.05
Pre-operation ESR (*X*_6_)					
< 15 mmol/h	(Control group)				
≥ 15 mmol/h	2.21	1.01	2.18	0.03	0.21~4.22
Primary disease (*X*_7_)					
KOA	(Control group)				
KRA	− 3.04	2.15	− 1.42	0.16	− 7.30~1.21
Merge disease (*X*_8_)					
No	(Control group)				
Yes	1.97	1.12	1.76	0.08	− 0.54~2.92
Tourniquet (*X*_9_)					
No	(Control group)				
Yes	8.60	4.58	1.88	0.06	− 0.48~17.68

*ASA* American Society of Anesthesiologists, *ESR* erythrocyte sedimentation, *KOA* knee osteoarthritis, *KRA* knee rheumatoid arthritis

## Discussion

Currently, ERAS program has performed well for patients after TKA. The ERAS program could decrease complications of surgery and shorten the LOS, further allowing patients to recover and discharge earlier. However, many factors may prolong the LOS in TKA patients, which are still unclear. Therefore, factors that caused the LOS prolongation in TKA patients should be identified to establish a better ERAS model. Most of the relevant researches were conducted in Europe and America, whereas Chinese patients with ERAS program were less covered [9]. Thus, it is of great significance to investigate the clinical situation of TKA patients with ERAS program in China and identify the influencing factors of LOS prolongation. In this study, the influencing factors of LOS were analyzed retrospectively to shorten LOS and optimize the ERAS model, further promoting the TKA surgery.

Many factors may influence the LOS in ERAS model, such as gender. Gender was reported to associate with knee osteoarthritis (KOA) [10–13]. The satisfaction rate of female patients was relatively low and the LOS was relatively long, compared with other patients. Our results suggested that female gender was a risk factor for LOS prolongation in the application of ERAS model. Compared with males, females had a higher probability of getting urinary tract infection and deep venous thrombosis [14, 15], further prolonging the LOS. Besides, the lower limb dysfunction and muscle weakness might lead to a slower postoperative rehabilitation, resulting in LOS extension. In the ERAS model, the orthopedic surgeon should pay more attention on the female patients and design the individualized therapy. Various interventions in the ERAS model might be effective to shorten the LOS. For example, strengthening the psychological education to the patient to reduce and eliminate their anxiety and fear, avoiding the retention catheterization and removing it in the early stage to prevent the urinary tract infection, adopting the combination therapy of physiotherapy and pharmacotherapy to avoid the deep venous thrombosis, and strengthening the early-stage rehabilitation exercise to promote the recovery of patients. All the strategies could optimize the ERAS model and shorten LOS.

ASA class indicates the risk of operation and anesthesia in patients. ASA class would affect the LOS, and the LOS would be prolonged half day per two levels of ASA class. If the ASA score was over 2, the probability of adverse events and the usage of opioid use would increase. Based on the ASA score, the anesthesiologist could optimize the anesthetic methods to shorten the LOS. Our study found that the higher ASA class (ASA class > 3) could increase the LOS. Thus, the orthopedic surgeon should evaluate the complexity of disease and risk of surgery during the perioperative period to identify the complicating disease and should do positive interventions. Besides, making appropriate anesthesia measures with the consultation of anesthetist and designing the relative prevention procedures could reduce stress response or complications in patients, which might be beneficial for recovery.

Nutrition support is one of the key elements in the ERAS model. The preoperative albumin reflects the nutrition status of patients, which participates in the promotion of incision healing. A high pre-operative Alb could promote the incision healing, while a low serum Alb would lead to a variety of complications or even death [16, 17]. Our study found that patients with preoperative Alb < 30 g/L had a longer LOS. Thus, the surgeon should pay more attention to the Alb of the patient and do periodical inspection. TKA can increase stress response and cause trauma, further leading to the impaired immunity and nutritional metabolism disorders [18]. Malnourished patients are too weak to overcome stress response, then, the recovery process will be slow and the LOS will be prolongated. Thus, during the perioperative period, the surgeon should consult with the nutritionist to assess the risk of malnutrition in patients and do the early intervention to assist the recovery of patients. Preoperative selective feeding, shortening the period of fasting, and encouraging early feeding after surgery would reduce the stress response and the possibility of complications. Besides, high-protein food could increase the protein levels in vivo and improve the incision healing. Maintaining the normal enteral nutrition could be effective. Combining the position adjustment and drug intervention could prevent the postoperative nausea and vomiting, further promoting the nutritional support.

Reducing inflammation and stress response are major parts of the ERAS model. ESR is a sensitive inflammatory indicator in serum, which can be used to predict the probability of infection. However, it cannot clearly indicate the clearance of the infection and direct the implant timing of the prosthesis [19–21]. Our study indicated that preoperative ESR ≥ 15 mm/h could lead to the prolongation of LOS. The surgeon should pay more attention to the ESR in patients and maintain it in the normal range. Strict aseptic operation and appropriate use of antibiotics could prevent infection. However, in clinical practice, we noticed that some patients’ ESR level were high before surgery and declined after surgery. This clinical phenomenon might be related with the primary diseases in patients, such as rheumatoid arthritis (RA). Thus, the surgeon should make individualized treatment schedule for patients with TKA, choose the surgery timing carefully, and strengthen the intervention in the perioperative period to shorten the LOS [22].

In addition, the operation time, primary diseases, and merge diseases might affect the LOS in patients. Sodhi et al. reported that the operation time was closely related to LOS [23]. Sizer et al. found that elder patients were easier to occur bleeding during operation and had increased complications [24]. Primary disease could affect patients’ satisfaction and the LOS [25]. Early postoperative rehabilitation could help to shorten the LOS in patients [26]. This paper reported that the above factors were significantly correlated with the LOS in TKA patients by the single-factor chi-square analysis (*P* < 0.05). However, the multi-factor linear regression analysis did not support the finding, and the significant association between the above factors and the LOS was above 0.05. This inconformity between the single-factor chi-square analysis and the multi-factor linear regression analysis might be explained by the limited sample amounts. We will increase the sample amounts and do the follow-up investigation in further research. Based on the current finding, we concluded that improving the surgical technique to shorten the time of operation, strengthening the hematology management to reduce the probability of bleeding and blood transfusion, enhancing the intervention of the active rehabilitation, and maintaining a regular follow-up might optimize the ERAS model and reduce the LOS.

## Conclusions

To sum up, LOS is related with patients’ satisfaction, while many factors might influence the LOS in TKA patients. Our study found that female, ASA class 3, preoperative albumin < 30 g/L, and preoperative ESR > 15 mmol/h were the risk factors to increase LOS. Some factors in this study did not appear to be statistically significant, which might be due to the small sample size. We will increase the sample size and follow-up on this study in the future. Our study suggests that the ERAS model should be optimized and designed on the individual’s status to shorten LOS in TKA patients.

## Data Availability

Please contact the author for data requests.

## Abbreviations

ASA: American Society of Anesthesiologists
BMI: Body mass index
CI: Confidence intervals
CRP: C-reactive protein
ERAS: Enhanced recovery after surgery
Hb: Hemoglobin
LOS: Length of stay
OA: Osteoarthritis
RA: Rheumatoid arthritis
TKA: Total knee arthroplasty
